# Nutrition policy critical to optimize response to climate, public health crises

**DOI:** 10.3389/fnut.2023.1118753

**Published:** 2023-08-16

**Authors:** Mark E. Rifkin

**Affiliations:** Center for Biological Diversity, Tucson, AZ, United States

**Keywords:** plant-based diet, health care capacity, chronic disease, nutrition policy, climate change, disasters and emergencies, public health, dietary change

## Abstract

The effects of unanticipated crises on health care and first-responder systems are reflected in climate-fueled environmental emergencies, to which human resilience is diminished by our chronic disease epidemic. For example, people who depend on specialized medications, like refrigerated insulin for diabetes, will likely face additional challenges in receiving treatment and care during extreme heat, floods, disasters, and other adverse events. These circumstances may be compounded by staff and equipment shortages, lack of access to fresh food, and inadequate healthcare infrastructure in the wake of a disaster. Simply put, our health care and first-response systems struggle to meet the demands of chronic disease without such crises and may be fundamentally unable to adequately function with such crises present. However, nutrition’s primacy in preventing and controlling chronic disease directly enhances individual and public resilience in the face of existential threats. Highlighting the shared diet-related etiology clearly demonstrates the need for a national policy response to reduce the disease burden and potentiate mitigation of the sequelae of climate risks and capacity limits in our food and health care systems. Accordingly, this article proposes four criteria for nutrition policy in the Anthropocene: objective government nutrition recommendations, healthy dietary patterns, adequate nutrition security, and effective nutrition education. Application of such criteria shows strong potential to improve our resiliency despite the climate and public health crises.

## Introduction

The American food system produces a gross imbalance of substantial overconsumption, undernutrition and food insecurity—prime contributors to an expanding chronic disease burden. Food system changes are also essential to meet climate reduction targets (1), but reform attempts have been inadequate. The disease burden is now compounded by climate-related impacts on disaster risk, food production and nutrition security, plus the COVID pandemic, producing a perfect storm threatening public health. The urgency of reforms in food policy, food production and eating patterns cannot be overstated. This paper highlights how nutrition policy changes critical to addressing the chronic disease epidemic could simultaneously mitigate climate-related risks and capacity limitations to individuals and the health care and food systems. This paper proposes four criteria essential for sound federal nutrition policy in the Anthropocene: objective government nutrition recommendations, a coordinated strategy to support healthy dietary patterns, adequate nutrition security, and effective, tailored nutrition education.

## Background

### Health care vulnerabilities in natural disasters

Climate-related temperature increases are expected to exacerbate extremes of precipitation, storms, hurricanes, floods, droughts, heat, and wildfires, and contribute to substantial health risks (2). In addition to direct harms, ensuing risks, including power outages, population displacement, healthcare facility evacuations and disruption of health care services can directly threaten health and lives. Subsequent poor health outcomes include aggravation of existing chronic diseases and conditions (2), often because of lost access to medications, providers (3), and general care and treatment (4).

Consecutive natural disasters^1^ are occurring more frequently (5), multiplying their immediate effects on public health and the capacity of first responders (5), affecting life and welfare and the capacity of key infrastructure and institutions. Delays in recovery from an initial disaster can have significant implications for future vulnerability (5), as observed in Puerto Rico, which was still recovering from multiple hurricanes in 2017 when Hurricane Fiona struck in 2022 (6). Consecutive disasters increase vulnerability of people, structures, and institutions to subsequent disasters; and the capabilities of health care and emergency responders stretched by the first event are further limited by a second event, increasing impacts synergistically. The complexities of addressing consecutive disaster risk have created significant barriers to effective policy solutions (5).

Hospitals routinely serve essential roles, including as frontline public health surveillance infrastructure. However, many hospitals also routinely approach maximum patient capacity during daily operations. Addition of a disaster produces an immediate increase in demand by those directly harmed or forced to evacuate without essential medications or home health equipment (7). Emergency events may also generate indirect and long-term consequences for facility-based health care and essential medical equipment and supplies. This diminishes capacity to accommodate any further increase in patient load, aka “surge capacity.” During evacuation or demand surges, systems operating near maximum capacity or serving high-need populations are particularly at risk (e.g., those with chronic illness or equity disparities). Health care facilities may also suffer structural damage, loss of potable water sources, and disorder in supply chains for critical equipment and supplies (3). Health care systems also generally lack sufficient resources to evaluate facility vulnerability in preparation for climate-related disasters (2).

The effects of healthcare facility power outages may be uniquely severe and require evacuation, which brings its own risks to medically vulnerable or temperature-sensitive patients during weather extremes. Additionally, the higher levels of care typical of modern medicine generally rely on electricity-dependent services and support systems (8).

Despite broad recognition of the critical nature of these issues, challenges maintaining emergency power in healthcare facilities continue. These may originate from common limitations of emergency standby systems, including poor design and maintenance, and an inability to withstand adverse conditions or automatically switch power sources as required (8). Since regulations only require emergency systems to meet power needs for essential functions, capacity of standby systems do not often exceed that minimum. In particular, air conditioning is often unsupported due to its huge power demand, despite risks of heat extremes (8). Other systems often omitted are the morgue, lab, blood bank, tissue storage, diagnostic equipment, medication dispensing systems, ventilation for computer server rooms, food storage, and elevators, each of which may become especially critical during emergencies. Fuel supplies required to operate backup systems may degrade in storage, and the disaster may affect re-supply. Additionally, about 15% of backup generators fail after 24 h of continuous use (8), which limits their utility amid such circumstances.

Accordingly, hospitals may struggle to maintain sufficient, reliable emergency power during outages, which increases challenges in caring for patients and may diminish quality of care. Consequently, inadequate emergency planning creates added risk to patients and further exacerbates equipment needs in a rescue or response scenario (8).

Emergency departments, which are routinely overcrowded, may be particularly vulnerable to the effects of disaster-related demand surges (3). More than 90% of U.S. emergency departments reported being overextended beyond maximum capacity at some point during normal operations, even pre-COVID (9). Existing shortages in medical and first responder staff (10–14) will only aggravate system vulnerabilities. One Michigan first responder stated, “If we do not figure out a way to build a stronger [first responder] foundation, it’s going to collapse… people had better pay attention to it before someone dials 911 and there’s nobody to respond” (15).

### Burden of diet-related chronic disease

Substantial health care resources are associated with treatment for obesity, diabetes, heart disease, cancer and hypertension (16, 17). Sixty percent of Americans now have at least one chronic disease, and 67% of that population (or 40% of the total) have at least two (18), nearly double that of 20 years ago (19). Additionally, health care needs tend to increase proportionally with population aging (20–23). Multiple dietary risk factors contribute to these diseases, including high intakes of animal-based foods, refined and processed foods, sodium, and saturated and trans fats, as well as insufficient intakes of fruits, vegetables, whole grains, beans, nuts and seeds and their important components, such as fiber and potassium (24).

The health benefits of plant-based diets accrue via multiple pathways. For example, the high content of soluble and insoluble fibers and phytonutrients and low content of saturated and trans fats slows digestion, stabilizes blood lipids and glucose, and supports a healthy microbiome, reducing risks for overweight, diabetes and cardiovascular disease (25). The higher content of potassium and other vaso-dilators combined with a lower content of sodium and oxidative components support healthy blood pressure (26). See Table 1 itemizing multiple relationships between plant foods and cardiovascular risk factors. Due to their therapeutic potential, plant-based dietary patterns have been recommended as primary therapy for diabetes, cardiovascular disease, hypertension, and obesity (32). The high fiber and phytonutrient content also facilitates cancer prevention, leading the American Cancer Society to recommend a protective dietary pattern focused on plant foods, especially deep-color fruits and vegetables and whole grains and legumes (33).

**Table 1:** Potential cardiovascular benefits of healthy plant foods.

	Fruit	Vegetables	Whole grains	Beans/Legumes	Nuts and seeds	Soy foods
Increase anti-oxidant activity	✔	✔	✔	✔	✔	✔
Inhibit platelet aggregation	✔	✔	✔	✔	✔	
Increase endothelial function	✔	✔	✔	✔	✔	
Reduce serum cholesterol	✔	✔	✔	✔	✔	✔
Inhibit serum LDL-C oxidation	✔	✔	✔	✔	✔	✔
Reduce blood pressure	✔	✔	✔	✔	✔	✔
Reduce serum triglycerides					✔	

Cardiovascular benefits of plant foods generally accrue via multiple components, including vitamins C and E, fiber, phytates, sterols, isoflavones, omega-3 fatty acids and various phytonutrients such as carotenoids, flavonoids, and polyphenols (27–31). These benefits may not apply to each member of each indicated food group or sub-group.

People with diabetes may be especially at risk during a natural disaster. Evacuation may alter dietary habits, force loss of suitable insulin storage, and interfere with access to medications and essential supplies. Such losses can disrupt medication compliance, which can alter blood glucose status and quickly produce life-threatening consequences (34, 35). After Hurricane Sandy, diabetes-related emergency department visits increased 84% (36).

### Synergistic effects of chronic disease and climate change on marginalized populations

Temperature extremes can increase morbidity and mortality associated with common chronic diseases and conditions, including cardiovascular disease, chronic kidney disease, diabetes, and hypertension (3, 37), as well as overweight and obesity (38). Food insecurity, another health risk factor, now affects more than 10% of Americans (39). Because of inadequate access to culturally-appropriate health services (40) and healthy food (41–43), as well as a disproportionate pollution burden (44), these diseases and conditions are highly prevalent in underserved, marginalized, and minority populations (40, 45). These communities, which are often forced to depend on emergency departments even for routine health care, are unlikely to receive adequate treatment during disasters and extreme weather events (3).

Chronic disease risks and food insecurity are also related to physical difficulties in performing daily activities (46). One in seven Americans have both a chronic disease and a functional limitation (47). People with functional limitations and the more general “access and functional needs^2^” (8) constitute about 30 to 50% of the population and will likely require special assistance during and after an emergency (8). However, standard preparedness and response planning often assumes adults are fully functional, mobile and capable of self-care, including in sub-optimal circumstances (48).

### Pandemic implications and risks

As observed during the COVID pandemic, emergencies can devastate supply chains and increase food insecurity, especially among women, minority and indigenous groups, immigrants, single parent households, and low-income groups (49). The COVID pandemic also added extraordinary burdens to the already overwhelmed health care system, including rapid increases in complex patient loads, utilization of intensive care systems, staff stress (50), and service demand beyond facility capacity (51). Moreover, the underlying burden of diet-related chronic disease directly increased COVID-related morbidity and mortality (52, 53). Minimizing the risks and effects of future pandemics is critical for optimal health care system function (54).

The scenario is further complicated by substantial evidence linking COVID and future pandemic risks to climate-induced changes on the global food system. Reduced agricultural production has driven the increased exploitation of forests, wildlife and habitat, which facilitates inter-species virus transmission (55–57). Pandemic risks also increase with global expansion of animal agriculture to meet rising demand for meat (58).

## Gaps observed

Gaps in policy, capacity, and key infrastructure and institutions include:

- Given the connection between largely-preventable chronic disease and individual and public resilience to the climate crisis, gaps exist in policy development and implementation to shift national dietary habits and improve nutrition and public health.
- Given the burdens of routine patient demand amid shortages in medical and responder staffing, gaps exist in the structural and surge capacity of healthcare facilities to accommodate climate or disaster shocks, especially if they are simultaneous.
- Given the high prevalence of chronic disease and infirmities, especially in marginalized communities, as well as health care access inequities and assumptions in response planning, gaps exist in the structural, economic and policy settings to mitigate the high prevalence of these chronic diseases.

**Figure 1:**
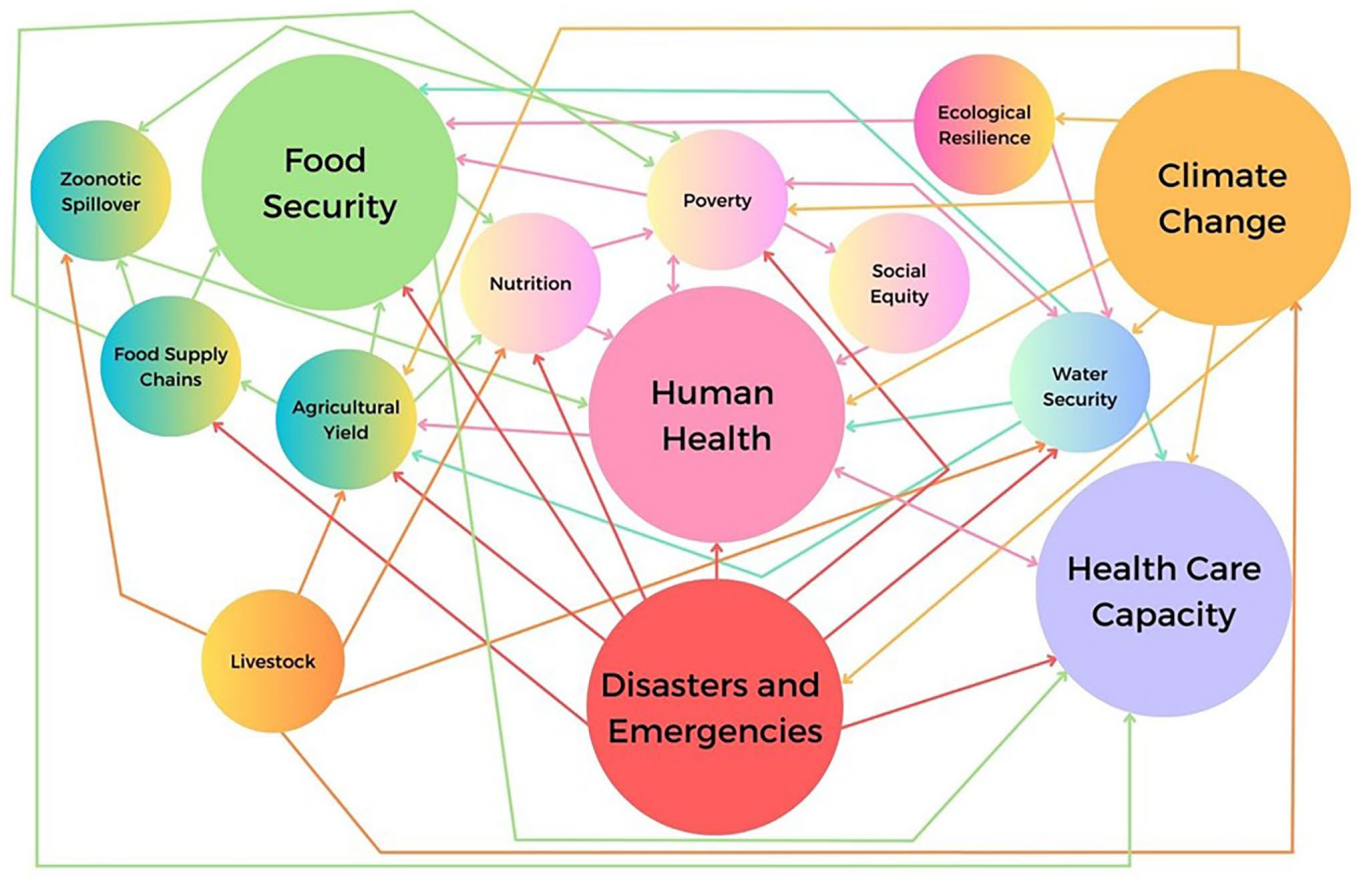
Public health systems map: interactions between food security, climate change and human health. Directional relationships between public health systems components are shown. Note some relationships are bi-directional. Also note the multiple impacts to human health and health care capacity.

These impacts are represented by the public health system map shown in Figure 1.

## Discussion: the potential of dietary change

The chronic disease burden is partly explained by the poor compliance with the Dietary Guidelines for Americans (59), and that most adults tend to overestimate their diet quality, sometimes by substantial margins (60, 61). Many consumers do not possess sufficient nutrition and health literacy to assess nutrient content from food labels (62). Individual lifestyles, family preferences, cultural and social norms, food accessibility and availability, time pressures, and personal cooking skills also influence nutrition-related decisions (63). Collectively, this suggests a need for nutrition education to help consumers customize healthy dietary patterns and clarify nutrition’s role in disease prevention. Widespread dietary change that effectively improves chronic disease risk factors reduces the disease burden, thereby optimizing individual and national resilience to climate and public health crises.

However, current dietary recommendations fail to produce meaningful changes in consumer food and nutrition behaviors toward the food groups that actively prevent disease, namely fruits, vegetables, whole grains, beans/legumes and nuts and seeds (64), indicating that dietary recommendations must be strengthened. The optimal solution may be plant-based dietary patterns, which emphasize plant protein and other healthy plant foods while reducing consumption of animal-based foods. As shown by Table 1, plant-based diets substantially reduce risks for chronic disease and hospitalization by optimizing intake of preventive and therapeutic dietary components while minimizing intake of dietary risk factors (65, 66). Such diets could also meaningfully modify climate-related (1) and zoonotic disease risks by reducing livestock production (58).

## Recommendations: the potential of policy change

Climate and public health threats demand multi-faceted, inter-disciplinary solutions, including structural changes which may require significant timelines. However, deliberate efforts to shift nutrition policy can drive shifts in dietary patterns over shorter timelines, which can then mitigate both health and climate risks. Because nutrition policy influences food availability, cost, accessibility, and access to health and nutrition expertise, strategic, inventive, and collaborative policy shifts can inspire behavior change. Policymakers, food industry leaders, institutions, and the public share a responsibility to create, promote, and implement effective nutrition policy that facilitates access to culturally-appropriate food and makes healthy foods the default option. Such policy change is mandatory to protect health and life amidst the existential risks we face. The following four criteria provide a pathway to policy change:

### Formalize objective nutrition recommendations

Congress and federal agencies must prioritize scientific evidence in implementing food and nutrition policy and programs while excluding undue industry influence. Industry influence increases doubt and confusion over direct, well-established food-and-disease relationships and impedes efforts to change dietary behaviors, allowing chronic diseases, zoonoses risks, climate change, disaster and emergency threats and other environmental crises to advance for the sake of corporate profit. Program examples include school meals, food and nutrition support programs, and community outreach and education programs. Such precepts should also apply to the Dietary Guidelines for Americans, the subject of significant concerns regarding undue political influence (67). After a thorough review, the National Academies of Sciences, Engineering and Medicine concluded “the adoption and widespread translation of the [Guidelines] require that they be universally viewed as valid, evidence based, and free of bias and conflicts of interest to the extent possible” (68). Therefore, agencies coordinating any food and nutrition program must commit to objectivity and transparency and avoid even the appearance of impropriety.

### Develop a coordinated nutrition strategy to support healthy dietary patterns

Currently, nutrition-related research and policy programs are divided among multiple departments and agencies with minimal coordination. Such disjointed programming affects nutrition policy, as well as health, agriculture and environmental protection. At the request of Congress, the Government Accountability Office issued a report recommending a coordinated effort to improve nutrition and chronic disease prevention (69). For example, it specifically addressed the inconsistency between federal subsidies that incentivize the use of controversial corn sweeteners while other agencies recommend limiting sweetener intake due to excessive chronic disease risk (70). Nutrition advocates support a coordinated federal interagency nutrition strategy (71), which could reduce program contradictions, advance nutrition research, and implement consistent, culturally-appropriate public communications. Such a strategy could also validate and incentivize effective behavior modification efforts to increase healthy food intake.

### Enhance food security

Advancing healthy dietary behaviors requires adjusting federal policy to prioritize improved food and nutrition security. For example, the president could order (1) a review of food and nutrition-related programs for adverse impacts to food and nutrition security (e.g., via climate change’s impacts on food production), (2) actions to prioritize funding toward smaller, independent farmers of color, and (3) actions to implement universal school meals, including plant-based menu options. By improving both food and nutrition security, these actions could reduce chronic disease risks and optimize the health and welfare of millions, ultimately improving resilience of high-risk groups to climate and disaster threats.

### Establish and support tailored nutrition education

Americans’ low nutrition literacy inhibits adoption of healthy dietary behaviors, indicating that federal policy must ensure culturally appropriate nutrition education is provided in schools (72). Additionally, the Centers for Disease Control and Prevention could implement public health education campaigns, as previously accomplished (73, 74), while the Department of Agriculture could expand existing nutrition education and outreach programs. Together these efforts could substantially improve dietary behaviors and reduce chronic disease risks. Finally, policy could address the near-total absence of nutrition content in medical school curricula, which minimizes competence necessary to provide even basic nutrition counseling (75). The absence of formal nutrition education policy constitutes a default decision to foster continued nutrition illiteracy and manipulation of the public by self-interested industries, facilitating continued increases in the severity of the chronic disease burden and further declines in individual resilience in climate and public health crises.

In conclusion, the threats represented by the concurrent crises of chronic disease, health care challenges, and climate change present fundamental and dangerous risks to health and life. Policymakers should recognize the next pandemic or climate-related disaster is imminent and seize the opportunity to modify the risks where possible. While improving our poor dietary habits is a significant challenge, it is a solvable problem.

## Data availability statement

The original contributions presented in the study are included in the article/supplementary material, further inquiries can be directed to the corresponding author.

## Author contributions

MR conceived and designed the article, conducted the research review, wrote the first draft, revised the manuscript, and approved the submitted version.

## Conflict of interest

The author declares that the research was conducted in the absence of any commercial or financial relationships that could be construed as a potential conflict of interest.

## Publisher’s note

All claims expressed in this article are solely those of the authors and do not necessarily represent those of their affiliated organizations, or those of the publisher, the editors and the reviewers. Any product that may be evaluated in this article, or claim that may be made by its manufacturer, is not guaranteed or endorsed by the publisher.

## Footnotes

^1^Consecutive disasters are defined as two or more events with direct impacts that overlap both spatially and temporally (that is, later events occur before recovery of the initial event(s) is completed).

^2^People with access and functional needs are defined as individuals with "disabilities, older adults, and individuals with limited English proficiency, limited access to transportation, and/or limited access to financial resources to prepare for, respond to, and recover from the emergency," among other types of needs. This term includes the 20% of the population with disabilities.

## References

[1] Clark MA Domingo NG Colgan K Thakrar SK Tilman D Lynch J. Global food system emissions could preclude achieving the 1.5 and 2 C climate change targets. Science. (2020) 370:705–8. doi: 10.1126/science.aba7357, PMID: 33154139

[2] Runkle J Svendsen ER Hamann M Kwok RK Pearce J. Population health adaptation approaches to the increasing severity and frequency of weather-related disasters resulting from our changing climate: a literature review and application to Charleston, South Carolina. Curr Environ Health Rep. (2018) 5:439–52. doi: 10.1007/s40572-018-0223-y, PMID: 30406894 PMC6472270

[3] Sorensen CJ Salas RN Rublee C Hill K Bartlett ES Charlton P. Clinical implications of climate change on US emergency medicine: challenges and opportunities. Ann Emerg Med. (2020) 76:168–78. doi: 10.1016/j.annemergmed.2020.03.010, PMID: 32507491

[4] Paterson DL Wright H Harris PN. Health risks of flood disasters. Clin Infect Dis. (2018) 67:1450–4. doi: 10.1093/cid/ciy227 30986298

[5] de Ruiter MC Couasnon A van den Homberg MJ Daniell JE Gill JC Ward PJ. Why we can no longer ignore consecutive disasters. Earth Fut. (2020) 8:e2019EF001425. doi: 10.1029/2019EF001425

[6] General Accountability Office. (2022). Hurricane recovery can take years—but for Puerto Rico, 5 years show its unique challenges. Available at: https://www.gao.gov/blog/hurricane-recovery-can-take-years-puerto-rico-5-years-show-its-unique-challenges

[7] Joy K. Ripple effect: how hurricanes and other disasters affect hospital care University of Michigan Lab Blog (2017) Available at: https://labblog.uofmhealth.org/industry-dx/ripple-effect-how-hurricanes-and-other-disasters-affect-hospital-care.

[8] Federal Emergency Management Agency. (2019). Healthcare facilities and power outages: Guidance for state, local, tribal, territorial, and private sector partners. Available at: https://www.fema.gov/sites/default/files/2020-07/healthcare-facilities-and-power-outages.pdf

[9] Kelen GD Wolfe R D’Onofrio G Mills AM Diercks D Stern SA. Emergency department crowding: the canary in the health care system. NEJM Catal Innov Care Deliv. (2021) Available at: https://catalyst.nejm.org/doi/full/10.1056/CAT.21.0217

[10] Basu S Berkowitz SA Phillips RL Bitton A Landon BE Phillips RS. Association of primary care physician supply with population mortality in the United States, 2005-2015. JAMA Intern Med. (2019) 179:506–14. doi: 10.1001/jamainternmed.2018.7624, PMID: 30776056 PMC6450307

[11] Kaiser Family Foundation. (2022). Primary care health professional shortage areas (HPSAs). Available at: https://www.kff.org/other/state-indicator/primary-care-health-professional-shortage-areas-hpsas/?currentTimeframe=0&sortModel= %7B%22colId%22:%22Population%20of%20Designated%20HPSAs%22,%22sort%22:%22desc%22%7D

[12] IHS Markit Ltd. The complexities of physician supply and demand: Projections from 2019 to 2034. Washington, DC: Association of American Medical Colleges (2021) Available at: https://www.aamc.org/media/54681/download.

[13] Juraschek SP Zhang X Ranganathan V Lin VW. Republished: United States registered nurse workforce report card and shortage forecast. Am J Med Qual. (2019) 34:473–81. doi: 10.1177/1062860619873217, PMID: 31479295

[14] Weixel N. (2021). Ambulance, EMT first responders face ‘crippling workforce shortage’. The Hill. Available at: https://thehill.com/regulation/labor/577879-ambulance-emt-first-responders-face-crippling-workforce-shortage/

[15] Shamus K. J. (2021). With EMS staffing crisis brewing for decades, COVID pushes shortage into danger zone. Yahoo News. Available at: https://news.yahoo.com/ems-staffing-crisis-brewing-decades-110032629.html

[16] Centers for Disease Control and Prevention. Health and economic costs of chronic diseases National Center for Chronic Disease Prevention and Health Promotion (2022) Available at: https://www.cdc.gov/chronicdisease/about/costs/.

[17] American Diabetes Association. Economic costs of diabetes in the U.S. in 2017. Diabetes Care. (2018) 41:917–28. doi: 10.2337/dci18-0007, PMID: 29567642 PMC5911784

[18] Centers for Disease Control and Prevention. Chronic diseases in America National Center for Chronic Disease Prevention and Health Promotion (2022) Available at: https://www.cdc.gov/chronicdisease/resources/infographic/chronic-diseases.htm.

[19] Boersma P Black L Ward B. Prevalence of multiple chronic conditions among US adults, 2018. Prev Chronic Dis. (2020) 17:200130:E106. doi: 10.5888/pcd17.200130, PMID: 32945769 PMC7553211

[20] Adhikari NK Fowler RA Bhagwanjee S Rubenfeld GD. Critical care and the global burden of critical illness in adults. Lancet. (2010) 376:1339–46. doi: 10.1016/S0140-6736(10)60446-1, PMID: 20934212 PMC7136988

[21] Samal L Fu HN Camara DS Wang J Bierman AS Dorr DA. Health information technology to improve care for people with multiple chronic conditions. Health Serv Res. (2021) 56:1006–36. doi: 10.1111/1475-6773.13860, PMID: 34363220 PMC8515226

[22] Gan TJ Scott M Thacker J Hedrick T Thiele RH Miller TE. American Society for Enhanced Recovery: advancing enhanced recovery and perioperative medicine. Anesth Analg. (2018) 126:1870–3. doi: 10.1213/ANE.0000000000002925 29624526

[23] Federal Interagency Forum on Aging-Related Statistics. Older Americans 2020: key indicators of well-being. Washington, DC: U.S. Government Printing Office (2020).

[24] Fanelli SM Jonnalagadda SS Pisegna JL Kelly OJ Krok-Schoen JL Taylor CA. Poorer diet quality observed among us adults with a greater number of clinical chronic disease risk factors. J Prim Care Community Health. (2020) 11:215013272094589. doi: 10.1177/2150132720945898 PMC7533933 32996366

[25] Trautwein EA McKay S. The role of specific components of a plant-based diet in management of dyslipidemia and the impact on cardiovascular risk. Nutrients. (2020) 12:2671. doi: 10.3390/nu12092671, PMID: 32883047 PMC7551487

[26] Joshi S Ettinger L Liebman SE. Plant-based diets and hypertension. Am J Lifestyle Med. (2020) 14:397–405. doi: 10.1177/1559827619875411, PMID: 33281520 PMC7692016

[27] Asgary S Rastqar A Keshvari M. Functional food and cardiovascular disease prevention and treatment: a review. J Am Coll Nutr. (2018) 37:429–55. doi: 10.1080/07315724.2017.1410867 29528772

[28] Panche AN Diwan AD Chandra SR. Flavonoids: an overview. J Nutr Sci. (2016) 5:e47. doi: 10.1017/jns.2016.41, PMID: 28620474 PMC5465813

[29] Slavin J. Whole grains and human health. Nutr Res Rev. (2004) 17:99–110. doi: 10.1079/NRR200374 19079919

[30] Pujol A Sanchis P Grases F Masmiquel L. Phytate intake, health and disease: “let thy food be thy medicine and medicine be thy food”. Antioxidants. (2023) 12:146. doi: 10.3390/antiox12010146, PMID: 36671007 PMC9855079

[31] Rodríguez L Mendez D Montecino H Carrasco B Arevalo B Palomo I. Role of *Phaseolus vulgaris* L. in the prevention of cardiovascular diseases—cardioprotective potential of bioactive compounds. Plan Theory. (2022) 11:186. doi: 10.3390/plants11020186, PMID: 35050073 PMC8779353

[32] Tuso PJ Ismail MH Ha BP Bartolotto C. Nutritional update for physicians: plant-based diets. Perm J. (2013) 17:61–6. doi: 10.7812/TPP/12-085 23704846 PMC3662288

[33] Rock CL Thomson C Gansler T Gapstur SM McCullough ML Patel AV. American Cancer Society guideline for diet and physical activity for cancer prevention. CA Cancer J Clin. (2020) 70:245–71. doi: 10.3322/caac.21591, PMID: 32515498

[34] Bogart T Nguyen E Owens C Robinson R. Creating a sustainable and reliable emergency preparedness program to promote appropriate health care resources use. Fed Pract. (2021) 38:154–9. doi: 10.12788/fp.0108, PMID: 34177219 PMC8221924

[35] Boulle P Kehlenbrink S Smith J Beran D Jobanputra K. Challenges associated with providing diabetes care in humanitarian settings. Lancet Diabetes Endocrinol. (2019) 7:648–56. doi: 10.1016/S2213-8587(19)30083-X, PMID: 30878269

[36] Al-Shihabi F Moore A Chowdhury TA. Diabetes and climate change. Diabet Med. (2022) 40:e14971. doi: 10.1111/dme.14971 36209378

[37] Ebi KL Capon A Berry P Broderick C de Dear R Havenith G. Hot weather and heat extremes: health risks. Lancet. (2021) 398:698–708. doi: 10.1016/S0140-6736(21)01208-3 34419205

[38] Koch CA Sharda P Patel J Gubbi S Bansal R Bartel MJ. Climate change and obesity. Horm Metab Res. (2021) 53:575–87. doi: 10.1055/a-1533-2861, PMID: 34496408 PMC8440046

[39] Coleman-Jensen A Rabbitt MP Gregory CA Singh A. Household food security in the United States in 2021. Economic research report 309 Economic Research Service (2022) Available at: https://www.ers.usda.gov/publications/pub-details/?pubid=104655.

[40] Centers for Disease Control and Prevention. (2022). Social determinants of health. Available at: https://www.cdc.gov/chronicdisease/programs-impact/sdoh.htm

[41] Centers for Disease Control and Prevention. (2022). What is health equity? Available at: https://www.cdc.gov/healthequity/whatis/index.html

[42] Seligman HK Laraia BA Kushel MB. Food insecurity is associated with chronic disease among low-income NHANES participants. J Nutr. (2010) 140:304–10. doi: 10.3945/jn.110.135764, PMID: 20032485 PMC2806885

[43] Bower KM Thorpe RJ Jr Rohde C Gaskin DJ. The intersection of neighborhood racial segregation, poverty, and urbanicity and its impact on food store availability in the United States. Prev Med. (2014) 58:33–9. doi: 10.1016/j.ypmed.2013.10.010, PMID: 24161713 PMC3970577

[44] Centers for Disease Control and Prevention (n.d.). Environmental justice dashboard. Available at: https://ephtracking.cdc.gov/Applications/ejdashboard/

[45] Centers for Disease Control and Prevention. (2021). Racism and health. Available at: https://www.cdc.gov/minorityhealth/racism-disparities/index.html

[46] Venci BJ Lee SY. Functional limitation and chronic diseases are associated with food insecurity among US adults. Ann Epidemiol. (2018) 28:182–8. doi: 10.1016/j.annepidem.2018.01.005, PMID: 29482742

[47] Alecxih L. Shen S. Chan I. Taylor D. (2010). Individuals living in the community with chronic conditions and functional limitations: a closer look. Report prepared for Office of the Assistant Secretary for Planning and Evaluation, Department of Health and Human Services at the Lewin group. Available at: https://aspe.hhs.gov/reports/individuals-living-community-chronic-conditions-functional-limitations-closer-look-0

[48] Ringel JS Chandra A Williams M Ricci KA Felton A Adamson DM. Enhancing public health emergency preparedness for special needs populations: a toolkit for state and local planning and response. Rand Health Q. (2011) 1:31 Available at https://www.rand.org/pubs/technical_reports/TR681.html 10.7249/rhq-01-03-19 PMC4945206 28083206

[49] Hunter L Gerritsen S Egli V. Changes in eating behaviours due to crises, disasters and pandemics: a scoping review. Nutr Food Sci. (2022) 53:358–90. doi: 10.1108/NFS-12-2021-0385

[50] Chang AY Cullen MR Harrington RA Barry M. The impact of novel coronavirus COVID-19 on noncommunicable disease patients and health systems: a review. J Intern Med. (2021) 289:450–62. doi: 10.1111/joim.13184, PMID: 33020988 PMC7675448

[51] Baxter D Casady CB. Proactive and strategic healthcare public-private partnerships (PPPs) in the coronavirus (COVID-19) epoch. Sustainability. (2020) 12:5097. doi: 10.3390/su12125097

[52] Dessie ZG Zewotir T. Mortality-related risk factors of COVID-19: a systematic review and meta-analysis of 42 studies and 423,117 patients. BMC Infect Dis. (2021) 21:855. doi: 10.1186/s12879-021-06536-3, PMID: 34418980 PMC8380115

[53] Mahamat-Saleh Y Fiolet T Rebeaud ME Mulot M Guihur A El Fatouhi D. Diabetes, hypertension, body mass index, smoking and COVID-19-related mortality: a systematic review and meta-analysis of observational studies. BMJ Open. (2021) 11:e052777. doi: 10.1136/bmjopen-2021-052777, PMID: 34697120 PMC8557249

[54] Anyfantakis D Mantadaki AE Mastronikolis S Spandidos DA Symvoulakis EK. COVID-19 pandemic and reasons to prioritize the needs of the health care system to ensure its sustainability: a scoping review from January to October 2020. Exp Ther Med. (2021) 22:1039–7. doi: 10.3892/etm.2021.10471, PMID: 34373725 PMC8343896

[55] Wegner GI Murray KA Springmann M Muller A Sokolow SH Saylors K. Averting wildlife-borne infectious disease epidemics requires a focus on socio-ecological drivers and a redesign of the global food system. eClinicalMedicine. (2022) 47:101386. doi: 10.1016/j.eclinm.2022.101386, PMID: 35465645 PMC9014132

[56] Bernstein AS Ando AW Loch-Temzelides T Vale MM Li BV Li H. The costs and benefits of primary prevention of zoonotic pandemics. Science. Advances. (2022) 8:eabl4183. doi: 10.1126/sciadv.abl4183, PMID: 35119921 PMC8816336

[57] Klous G Huss A Heederik DJ Coutinho RA. Human–livestock contacts and their relationship to transmission of zoonotic pathogens, a systematic review of literature. One Health. (2016) 2:65–76. doi: 10.1016/j.onehlt.2016.03.001, PMID: 28616478 PMC5462650

[58] Hayek MN. The infectious disease trap of animal agriculture. Sci Adv. (2022) 8:eadd6681. doi: 10.1126/sciadv.add6681, PMID: 36322670 PMC9629715

[59] Kuczynski K. Healthy eating index Center for Nutrition Policy and Promotion (2022) Available at: https://www.fns.usda.gov/healthy-eating-index-hei.

[60] Thomson J Landry A Walls T. Can United States adults accurately assess their diet quality? Curr Dev Nutr. (2022) 6:952–2. doi: 10.1093/cdn/nzac067.072 36325649

[61] Cheng J Costacou T Sereika SM Rockettewagner B Kriska AM Klem ML. Relationship between perceived and measured diet quality improvements in a randomized weight loss trial. Circulation. (2022) 146:A10130–07. doi: 10.1161/circ.146.suppl_1.10130#d9794260e115

[62] Persoskie A Hennessy E Nelson WL. Peer reviewed: US consumers’ understanding of nutrition labels in 2013: the importance of health literacy. Prev Chronic Dis. (2017) 14:E86. doi: 10.5888/pcd14.170066, PMID: 28957033 PMC5621522

[63] Webb D Byrd-Bredbenner C. Overcoming consumer inertia to dietary guidance. Adv Nutr. (2015) 6:391–6. doi: 10.3945/an.115.008441, PMID: 26178023 PMC4496743

[64] Springmann M Spajic L Clark MA Poore J Herforth A Webb P. The healthiness and sustainability of national and global food based dietary guidelines: modelling study. BMJ. (2020) 370:m2322. doi: 10.1136/bmj.m2322, PMID: 32669369 PMC7362232

[65] Craig WJ Mangels AR Fresán U Marsh K Miles FL Saunders AV. The safe and effective use of plant-based diets with guidelines for health professionals. Nutrients. (2021) 13:4144. doi: 10.3390/nu13114144, PMID: 34836399 PMC8623061

[66] Melina V Craig W Levin S. Position of the academy of nutrition and dietetics: vegetarian diets. J Acad Nutr Diet. (2016) 116:1970–80. doi: 10.1016/j.jand.2016.09.025, PMID: 27886704

[67] Corporate Accountability. (2020). Media brief: dietary guidelines for corporate America. Available at: https://www.corporateaccountability.org/resources/dietary-guidelines-corporate-america/

[68] National Academies of Sciences, Engineering, Medicine. Redesigning the process for establishing the dietary guidelines for Americans. Washington, DC: The National Academies Press (2017) Available at: https://nap.nationalacademies.org/catalog/24883/redesigning-the-process-for-establishing-the-dietary-guidelines-for-americans. 29232083

[69] The United States Government Accountability Office. (2021). Chronic health conditions: federal strategy needed to coordinate diet-related efforts. Available at: https://www.gao.gov/products/gao-21-593

[70] Know Your Limit for Added Sugars. (2022). Centers for Disease Control and Prevention. Available at: https://www.cdc.gov/healthyweight/healthy_eating/sugar.html

[71] Fleischhacker SE Woteki CE Coates PM Hubbard VS Flaherty GE Glickman DR. Strengthening national nutrition research: rationale and options for a new coordinated federal research effort and authority. Am J Clin Nutr. (2020) 112:721–69. doi: 10.1093/ajcn/nqaa179, PMID: 32687145 PMC7454258

[72] Hayes D Contento IR Weekly C. Position of the academy of nutrition and dietetics, society for nutrition education and behavior, and school nutrition association: comprehensive nutrition programs and services in schools. J Nutr Educ Behav. (2018) 50:433–439.e1. doi: 10.1016/j.jneb.2018.03.001, PMID: 29751854

[73] Centers for Disease Control and Prevention. (2022). Campaign resources. Available at: https://www.cdc.gov/tobacco/campaign/tips/resources/index.html

[74] Centers for Disease Control and Prevention. (2021). Communication resources. Available at: https://www.cdc.gov/coronavirus/2019-ncov/communication/index.html

[75] Logan AC Prescott SL Katz DL. Golden age of medicine 2.0: lifestyle medicine and planetary health prioritized. J Lifestyle Med. (2019) 9:75–91. doi: 10.15280/jlm.2019.9.2.75 31828026 PMC6894443

